# Crystal structure and Hirshfeld surface analysis of 2-oxo-2-phenyl­ethyl 3-nitroso-2-phenyl­imidazo[1,2-*a*]pyridine-8-carboxyl­ate

**DOI:** 10.1107/S2056989022001517

**Published:** 2022-02-15

**Authors:** Fouad El Kalai, Cemile Baydere, Necmi Dege, Abdulmalik Abudunia, Noureddine Benchat, Khalid Karrouchi

**Affiliations:** a Laboratory of Applied Chemistry and Environment (LCAE), Faculty of Sciences, Mohammed I University, 60000 Oujda, Morocco; b Department of Physics, Faculty of Arts and Sciences, Ondokuz Mayıs University, 55139-Samsun, Turkey; c Department of Pharmacology, Faculty of Clinical Pharmacy, University of Medical and Applied Sciences, Yemen; d Laboratory of Analytical Chemistry and Bromatology, Faculty of Medicine and Pharmacy, Mohammed V University in Rabat, Morocco

**Keywords:** crystal structure, hydrogen bonding, Hirshfeld surface analysis, imidazo[1,2-*a*]pyridine

## Abstract

The title compound, C_22_H_15_N_3_O_4_, is built up from a central imidazo[1,2-*a*]pyridine ring system connected to a nitroso group, a phenyl ring and a 2-oxo-2-phenyl­ethyl acetate group. The imidazo[1,2-*a*] pyridine ring system is almost planar (r.m.s. deviation = 0.017 Å) and forms dihedral angles of 22.74 (5) and 45.37 (5)°, respectively, with the phenyl ring and the 2-oxo-2-phenyl­ethyl acetate group. In the crystal, the mol­ecules are linked into chains parallel to the *b* axis by C—H⋯O hydrogen bonds, generating *R*_2_^1^ (5) and *R*_4_^4^ (28) graph-set motifs. The chains are further linked into a three-dimensional network by C—H⋯π and π-stacking inter­actions. The inter­molecular inter­actions were investigated using Hirshfeld surface analysis and two-dimensional fingerprint plots, revealing that the most important contributions for the crystal packing are from H⋯H (36.2%), H⋯C/C⋯H (20.5%), H⋯O/O⋯H (20.0%), C⋯O/O⋯C (6.5%), C⋯N/N⋯C (6.2%), H⋯N/N⋯H (4.5%) and C⋯C (4.3%) inter­actions.

## Abstract 2

In the crystal, mol­ecules are linked by C—H⋯O hydrogen bonds, generating 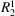 (5) and 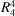 (28) ring motifs. In addition, weak C—H⋯π and π-stacking inter­actions are observed.

## Chemical context

Numerous drugs contain *N*-heterocycles as the core structure, including imidazo[1,2-*a*]pyridine and its derivatives, which are used in medicinal chemistry (Swainston Harrison & Keating, 2005 ▸; Deep *et al.*, 2017 ▸) or that exhibit diverse biological properties, such as anti­bacterial (Mishra *et al.*, 2021 ▸), anti­tubercular (Wang *et al.*, 2019 ▸), tyrosinase inhibitory (Damghani *et al.*, 2020 ▸), HIV inhibitory (Bode *et al.*, 2011 ▸), anti­diabetic (Saeedi *et al.*, 2021 ▸), anti-inflammatory (Gundlewad *et al.*, 2020 ▸) or anti­cancer activities (Yu *et al.*, 2020 ▸; Sigalapalli *et al.*, 2021 ▸). Encouraged by these features and in a continuation of our exploration of the synthesis, mol­ecular structures and Hirshfeld surface analysis of new *N*-heterocyclic compounds (Daoui *et al.*, 2021 ▸, 2022 ▸; El Kalai *et al.*, 2021*a* ▸,*b* ▸), we report herein the crystal structure and Hirshfeld surface analysis of 2-oxo-2-phenyl­ethyl 3-nitroso-2-phenyl­imidazo[1,2-*a*]pyridine-8-carboxyl­ate, C_22_H_15_N_3_O_4_ (I). 
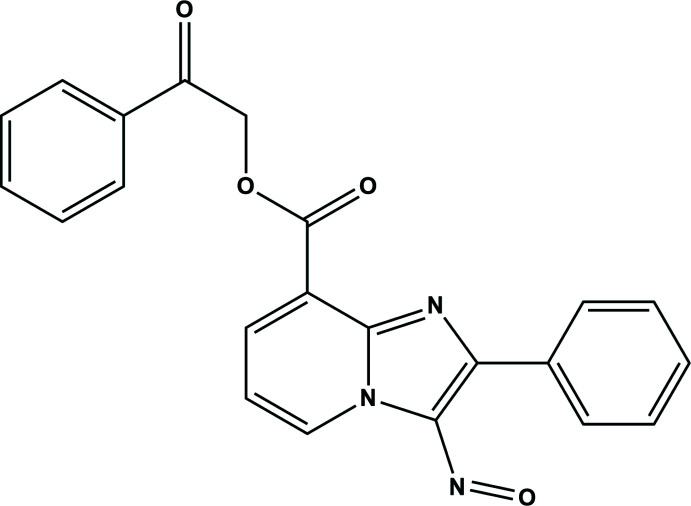


## Structural commentary

**Figure 1:**
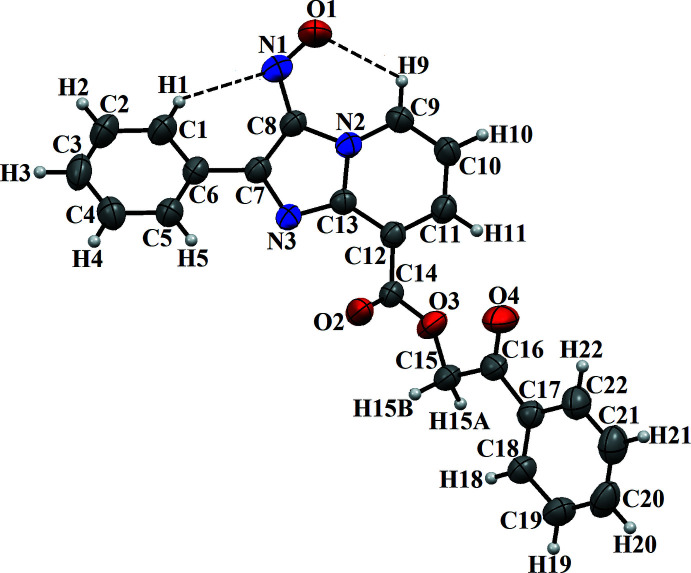
The mol­ecular structure of (I), with atom labelling. Displacement ellipsoids are drawn at the 50% probability level. Intra­molecular hydrogen bonds are indicated by dashed lines.

The mol­ecular structure of (I) is shown in Fig. 1 ▸. The imidazo[1,2-*a*] pyridine ring system is planar with an r.m.s deviation of 0.017 Å and a maximum deviation of 0.028 (1) Å for atom C11. The mean plane through the fused ring system makes dihedral angles of 22.74 (5) and 45.37 (5)° with the phenyl ring (C1–C6) and the 2-oxo-2-phenyl­ethyl acetate group (C14–C22), respectively. The dihedral angle between the two aromatic rings (C1–C6 and C17–C22) is 59.63 (5)°. The mol­ecular conformation is stabilized by two weak intra­molecular C9—H9⋯O1 and C1—H1⋯N1 hydrogen bonds, generating *S*(6) ring motifs (Table 1 ▸, Fig. 1 ▸).

## Supra­molecular features

**Figure 2:**
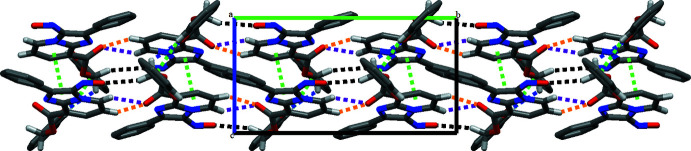
A view along the *a* axis of the crystal structure of (I). Blue, black, purple and orange dashed lines symbolize inter­molecular C15—H15*A*⋯O4^i^, C15—H15*B*⋯O1^ii^, C9—H9⋯O2^iii^ and C10—H10⋯O2^iii^ hydrogen bonds, respectively; π–π and C—H⋯π inter­actions are shown as green dashed lines.

In the crystal, mol­ecules are linked by C9—H9⋯O2^iii^ and C10—H10⋯O2^iii^ hydrogen bonds, forming chains that propagate parallel to the *b* axis and enclose 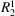 (5) ring motifs (Table 1 ▸, Fig. 2 ▸). Additionally, inter­molecular C15—H15*A*⋯O4^i^ and C15—H15*B*⋯O1^ii^ hydrogen bonds with 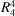 (28) ring motifs are also present, generating a three-dimensional supra­molecular network that also comprises a weak C22—H22⋯*Cg*4^iv^ inter­action (*Cg*4 is the centroid of the C17–C22 phenyl ring) as well as π–π stacking inter­actions involving the centroids (*Cg*1 and *Cg*2) of the N2/C13/N3/C7–C8 and N2/C9–C13 rings with a centroid-to-centroid distance *Cg*1⋯*Cg*2 (x, 1/2 − y, −1/2 + z) of 3.5750 (9) Å and a slippage of 0.685 Å (Fig. 2 ▸).

## Database survey

A search of the Cambridge Structural Database (CSD, version 5.40, update of August 2019; Groom *et al.*, 2016 ▸) using 2-phenyl­imidazo[1,2-*a*]pyridin-3-amine as the main skeleton revealed the presence of 54 structures with different substit­uents on the imidazo[1,2-*a*]pyridine ring. The two structures most similar to (I) are *N*-(2-phenyl­imidazo[1,2-*a*]pyridin-3-yl)acetamide (MIXZOJ; Anaflous *et al.*, 2008 ▸) and 4-[(7-methyl-2-phenyl­imidazo[1,2-*a*]pyridin-3-yl)carbonoimido­yl]phenol (TUQCEP; Elaatiaoui *et al.*, 2015 ▸). In MIXZOJ, C_15_H_13_N_3_O, the crystal structure consists of mol­ecular columns that are inter­connected by N—H⋯N hydrogen bonds along the *b-*axis direction. The torsion angle between the imidazo[1,2-*a*]pyridine ring system and the phenyl ring is 9.04 (5)°. In TUQCEP, C_21_H_17_N_3_O, the fused ring system is almost planar (r.m.s. deviation = 0.031 Å) and forms dihedral angles of 64.97 (7) and 18.52 (6)° with the phenyl ring and the (imino­meth­yl)phenol group, respectively. In its crystal, mol­ecules are linked by pairs of C—H⋯π inter­actions into centrosymmetric dimeric units, which are further connected by O—H⋯N hydrogen bonds, forming layers parallel to (101).

## Hirshfeld surface analysis

**Figure 3:**
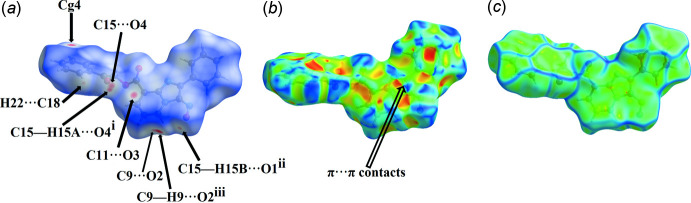
(*a*) *d*_norm_ mapped on the Hirshfeld surface to visualize the inter­molecular inter­actions, (*b*) shape-index map of the title compound and (*c*) curvedness map of the title compound using a range from −4 to 4 Å.

**Figure 4:**
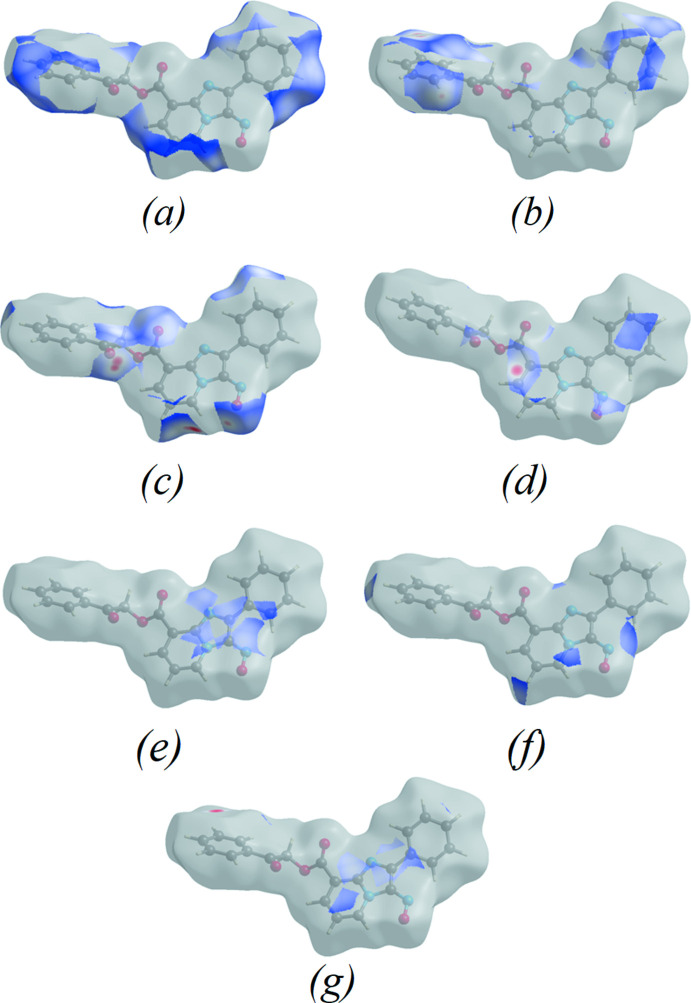
The Hirshfeld surface representations of (**I**) with the function *d*_norm_ plotted onto the surface for (*a*) H⋯H, (b) H⋯C/C⋯H, (*c*) H⋯O/O⋯H, (*d*) C⋯O/O⋯C, (*e*) C⋯N/N⋯C, (*f*) H⋯N/N⋯H and (*g*) C⋯C inter­actions.

**Figure 5:**
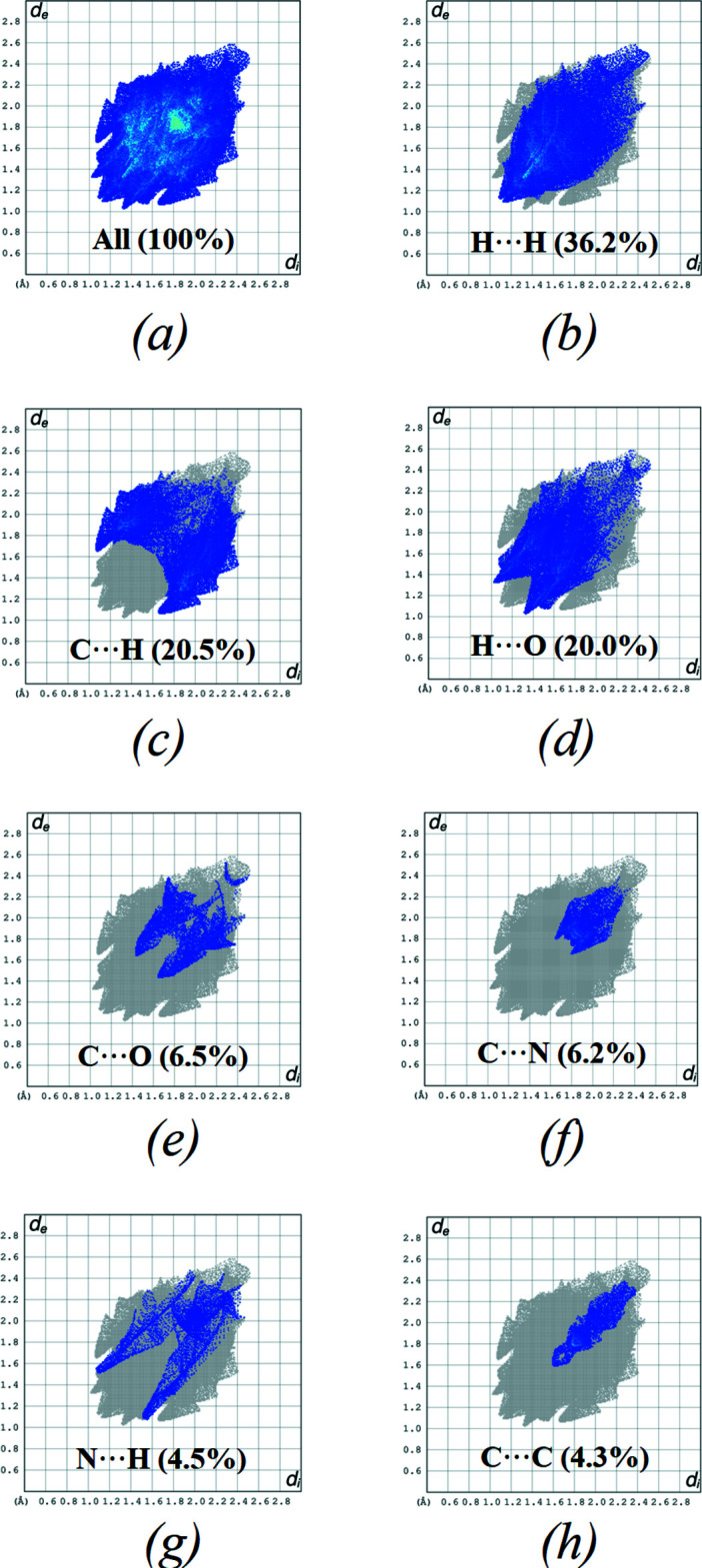
The full two-dimensional fingerprint plots for the title compound, showing (*a*) all inter­actions, and delineated into (*b*) H⋯H, (*c*) H⋯C/C⋯H, (*d*) H⋯O/ O⋯H, (*e*) C⋯O/O⋯C, (*f*) C⋯N/N⋯C, (*g*) H⋯N/N⋯H and (*h*) C⋯C inter­actions, together with their relative contributions.

Hirshfeld surface analysis was used to qu­antify the inter­molecular contacts of the title compound, using *Crystal Explorer* (Turner *et al.*, 2017 ▸). The Hirshfeld surface was generated with a standard (high) surface resolution and with the three-dimensional *d*_norm_ surface plotted over a fixed colour scale of −0.1706 (red) to 1.2371 (blue) a.u. (Fig. 3 ▸ *a*). The shape-index map of the title mol­ecule was generated in the range −1 to 1 Å (Fig. 3 ▸ *b*), revealing the presence of red and blue triangles that are indicative of the presence of π–π stacking inter­actions. The curvedness map of the title complex was generated in the range −4.0 to 4.0 Å (Fig. 3 ▸ *c*) and shows flat surface patches characteristic of planar stacking. The Hirshfeld surface representations with the function *d*_norm_ plotted onto the surface are shown for the H⋯H, H⋯C/C⋯H, H⋯O/O⋯H, C⋯O/O⋯C, C⋯N/N⋯C, H⋯N/N⋯H and C⋯C inter­actions in Fig. 4 ▸ *a–g*, respectively. The overall two-dimensional fingerprint plot is illustrated in Fig. 5 ▸ *a*, with those delineated into H⋯H, H⋯C/C⋯H, H⋯O/O⋯H, C⋯O/O⋯C, C⋯N/N⋯C, H⋯N/N⋯H and C⋯C contacts associated with their relative contributions to the Hirshfeld surface in Fig. 5 ▸ *b*–*h*, respectively. The most important inter­molecular inter­action is H⋯H, contributing 36.2% to the overall crystal packing (Fig. 5 ▸ *b*). H⋯C/C⋯H contacts, with a 20.5% contribution to the Hirshfeld surface, indicate the presence of the weak C—H⋯π inter­action (Table 1 ▸). Two pairs of characteristic wings in the fingerprint plot with pairs of tips at *d*_e_ + *d*_i_ ∼2.74 Å are present (Fig. 5 ▸ *c*). H⋯O/O⋯H contacts arising from inter­molecular C—H⋯O hydrogen bonding make a 20.0% contribution to the Hirshfeld surface and are represented by a pair of sharp spikes in the region *d*_e_ + *d*_i_ ∼2.34 Å (Fig. 5 ▸ *d*). The C⋯C contacts are a measure of π–π stacking inter­actions and contribute 4.3% of the Hirshfeld surface (Fig. 5 ▸ *h*). The contributions of the other contacts to the Hirshfeld surface are C⋯O/O⋯C of 6.5%, C⋯N/N⋯C of 6.2% and H⋯N/N⋯H of 4.5%.

## Synthesis and crystallization

To a solution of 2-oxo-2-phenyl­ethyl 2-phenyl­imidazo[1,2-*a*]pyridine-8-carboxyl­ate (0.71 g, 2 mmol) in acetic acid (50 ml), sodium nitrite (1.4 g, 2 mmol) was added at room temperature. The resulting precipitate was washed with water and extracted with di­chloro­methane (3 × 20 ml). The combined di­chloro­methane extracts were dried over anhydrous sodium sulfate and filtered. The remaining solution was concentrated under reduced pressure. The residue was purified chromatographically on a neutral alumina gel column using di­chloro­methane as eluent. Single crystals were obtained by slow evaporation of a di­chloro­methane solution at room temperature (yield 80%).

## Refinement

Crystal data, data collection and structure refinement details are summarized in Table 2 ▸. Hydrogen atoms were fixed geometrically and treated as riding, with C—H = 0.97 Å for methyl­ene [*U*_iso_(H) = 1.5*U*_eq_(C)], C—H = 0.93 Å for aromatic [*U*_iso_(H) = 1.2*U*_eq_(C)] and C—H = 0.98 Å for methine [*U*_iso_(H) = 1.2*U*_eq_(C)] H atoms.

## Supplementary Material

Crystal structure: contains datablock(s) I. DOI: 10.1107/S2056989022001517/wm5632sup1.cif

Structure factors: contains datablock(s) I. DOI: 10.1107/S2056989022001517/wm5632Isup2.hkl

Supporting information file. DOI: 10.1107/S2056989022001517/wm5632Isup3.cml

CCDC reference: 2106558

Additional supporting information:  crystallographic information; 3D view; checkCIF report

## Figures and Tables

**Table 1:** Hydrogen-bond geometry (Å, °) *Cg*4 is the centroid of the C17–C22 phenyl ring.

*D*—H⋯*A*	*D*—H	H⋯*A*	*D*⋯*A*	*D*—H⋯*A*
C15—H15*A*⋯O4^i^	0.97	2.54	3.1257 (19)	119
C15—H15*B*⋯O1^ii^	0.97	2.61	3.4841 (18)	150
C9—H9⋯O2^iii^	0.93	2.46	3.1176 (16)	128
C10—H10⋯O2^iii^	0.93	2.67	3.2243 (17)	119
C9—H9⋯O1	0.93	2.35	2.8736 (18)	116
C1—H1⋯N1	0.93	2.51	3.081 (2)	120
C22—H22⋯*Cg*4^iv^	0.93	2.80	3.657 (2)	153

Symmetry codes: (i) 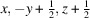 ; (ii) 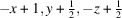 ; (iii) 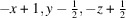 ; (iv) 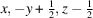 .

**Table 2:** Experimental details

Crystal data	
Chemical formula	C_22_H_15_N_3_O_4_
*M*_r_	385.37
Crystal system, space group	Monoclinic, *P*2_1_/*c*
Temperature (K)	296
*a*, *b*, *c* (Å)	15.9256 (14), 14.8256 (14), 7.6787 (6)
β (°)	90.566 (7)
*V* (Å^3^)	1812.9 (3)
*Z*	4
Radiation type	Mo *K*α
μ (mm^−1^)	0.10
Crystal size (mm)	0.56 × 0.38 × 0.15

Data collection	
Diffractometer	Stoe IPDS 2
Absorption correction	Integration (*X-RED32*; Stoe & Cie, 2012 ▸)
*T*_min_, *T*_max_	0.946, 0.969
No. of measured, independent and observed [*I* > 2σ(*I*)] reflections	27945, 6703, 3040
*R*_int_	0.070
(sin θ/λ)_max_ (Å^−1^)	0.765

Refinement	
*R*[*F* ^2^ > 2σ(*F* ^2^)], *wR*(*F* ^2^), *S*	0.046, 0.118, 0.92
No. of reflections	6703
No. of parameters	262
H-atom treatment	H-atom parameters constrained
Δρ_max_, Δρ_min_ (e Å^−3^)	0.15, −0.16

Computer programs: *X-AREA* and *X-RED* (Stoe & Cie, 2012 ▸), *SHELXT* (Sheldrick, 2015*a* ▸), *SHELXL* (Sheldrick, 2015*b* ▸), *Mercury* (Macrae *et al.*, 2020 ▸), *WinGX* (Farrugia, 2012 ▸), *PLATON* (Spek, 2020 ▸) and *publCIF* (Westrip, 2010 ▸).

## supplementary crystallographic information

### Crystal data

**Table:**

C_22_H_15_N_3_O_4_	*F*(000) = 800
*M**_r_* = 385.37	*D*_x_ = 1.412 Mg m^−^^3^
Monoclinic, *P*2_1_/*c*	Mo *K*α radiation, λ = 0.71073 Å
*a* = 15.9256 (14) Å	Cell parameters from 18578 reflections
*b* = 14.8256 (14) Å	θ = 1.9–32.8°
*c* = 7.6787 (6) Å	µ = 0.10 mm^−^^1^
β = 90.566 (7)°	*T* = 296 K
*V* = 1812.9 (3) Å^3^	Rod, green
*Z* = 4	0.56 × 0.38 × 0.15 mm

### Data collection

**Table:**

Stoe IPDS 2 diffractometer	6703 independent reflections
Radiation source: sealed X-ray tube, 12 x 0.4 mm long-fine focus	3040 reflections with *I* > 2σ(*I*)
Plane graphite monochromator	*R*_int_ = 0.070
Detector resolution: 6.67 pixels mm^-1^	θ_max_ = 32.9°, θ_min_ = 2.6°
rotation method scans	*h* = −24→24
Absorption correction: integration (*X-RED32*; Stoe & Cie, 2012)	*k* = −22→22
*T*_min_ = 0.946, *T*_max_ = 0.969	*l* = −10→11
27945 measured reflections	

### Refinement

**Table:**

Refinement on *F*^2^	0 restraints
Least-squares matrix: full	Hydrogen site location: inferred from neighbouring sites
*R*[*F*^2^ > 2σ(*F*^2^)] = 0.046	H-atom parameters constrained
*wR*(*F*^2^) = 0.118	*w* = 1/[σ^2^(*F*_o_^2^) + (0.0506*P*)^2^] where *P* = (*F*_o_^2^ + 2*F*_c_^2^)/3
*S* = 0.92	(Δ/σ)_max_ < 0.001
6703 reflections	Δρ_max_ = 0.15 e Å^−^^3^
262 parameters	Δρ_min_ = −0.16 e Å^−^^3^

### Special details

**Table:**

Geometry. All esds (except the esd in the dihedral angle between two l.s. planes) are estimated using the full covariance matrix. The cell esds are taken into account individually in the estimation of esds in distances, angles and torsion angles; correlations between esds in cell parameters are only used when they are defined by crystal symmetry. An approximate (isotropic) treatment of cell esds is used for estimating esds involving l.s. planes.

### Fractional atomic coordinates and isotropic or equivalent isotropic displacement parameters (Å^2^)

**Table:**

	*x*	*y*	*z*	*U*_iso_*/*U*_eq_	
O3	0.63005 (6)	0.31601 (7)	0.48667 (14)	0.0542 (3)	
O2	0.58609 (6)	0.39797 (6)	0.25605 (15)	0.0595 (3)	
N3	0.40012 (7)	0.34790 (7)	0.20621 (16)	0.0441 (3)	
N2	0.39287 (6)	0.19477 (7)	0.20006 (16)	0.0435 (3)	
O4	0.75836 (8)	0.26469 (8)	0.30406 (19)	0.0788 (4)	
O1	0.25639 (7)	0.09653 (7)	0.0672 (2)	0.0780 (4)	
N1	0.25076 (8)	0.18024 (9)	0.06361 (19)	0.0599 (4)	
C14	0.58089 (8)	0.33230 (9)	0.34708 (19)	0.0416 (3)	
C12	0.52135 (8)	0.25563 (8)	0.31845 (18)	0.0414 (3)	
C13	0.44102 (8)	0.27009 (8)	0.24261 (18)	0.0404 (3)	
C7	0.32472 (8)	0.32353 (9)	0.13986 (19)	0.0441 (3)	
C17	0.86457 (9)	0.34187 (9)	0.46238 (19)	0.0464 (3)	
C8	0.31681 (8)	0.22879 (9)	0.1315 (2)	0.0465 (3)	
C6	0.26278 (8)	0.39215 (9)	0.08286 (19)	0.0459 (3)	
C11	0.54667 (9)	0.16813 (9)	0.3493 (2)	0.0483 (3)	
H11	0.598339	0.157821	0.403177	0.058*	
C16	0.77582 (9)	0.32058 (9)	0.4145 (2)	0.0495 (3)	
C15	0.70537 (8)	0.36842 (9)	0.5070 (2)	0.0498 (3)	
H15A	0.719202	0.375040	0.629688	0.060*	
H15B	0.697194	0.428055	0.457752	0.060*	
C18	0.88553 (9)	0.40814 (10)	0.5833 (2)	0.0524 (4)	
H18	0.843669	0.443252	0.632786	0.063*	
C9	0.42025 (9)	0.10793 (9)	0.2257 (2)	0.0508 (4)	
H9	0.387103	0.059250	0.191712	0.061*	
C10	0.49660 (9)	0.09435 (9)	0.3016 (2)	0.0529 (4)	
H10	0.515667	0.035964	0.321857	0.063*	
C5	0.28989 (9)	0.47829 (10)	0.0411 (2)	0.0537 (4)	
H5	0.346539	0.492649	0.052027	0.064*	
C4	0.23357 (10)	0.54324 (11)	−0.0168 (2)	0.0618 (4)	
H4	0.252468	0.600751	−0.044788	0.074*	
C19	0.96856 (10)	0.42184 (11)	0.6299 (3)	0.0645 (4)	
H19	0.982383	0.465939	0.711376	0.077*	
C3	0.14938 (11)	0.52236 (12)	−0.0328 (2)	0.0669 (5)	
H3	0.111528	0.565477	−0.073272	0.080*	
C1	0.17725 (9)	0.37247 (11)	0.0689 (3)	0.0633 (4)	
H1	0.157736	0.315422	0.098519	0.076*	
C22	0.92826 (10)	0.29109 (11)	0.3880 (2)	0.0639 (4)	
H22	0.914895	0.247016	0.306093	0.077*	
C20	1.03132 (10)	0.37028 (13)	0.5559 (3)	0.0719 (5)	
H20	1.087105	0.379435	0.588214	0.086*	
C2	0.12162 (10)	0.43762 (13)	0.0113 (3)	0.0727 (5)	
H2	0.064726	0.424057	0.002223	0.087*	
C21	1.01088 (11)	0.30541 (13)	0.4343 (3)	0.0736 (5)	
H21	1.053005	0.271212	0.383457	0.088*	

### Atomic displacement parameters (Å^2^)

**Table:**

	*U*^11^	*U*^22^	*U*^33^	*U*^12^	*U*^13^	*U*^23^
O3	0.0450 (5)	0.0615 (6)	0.0559 (7)	−0.0123 (4)	−0.0143 (5)	0.0119 (5)
O2	0.0558 (6)	0.0431 (5)	0.0792 (8)	−0.0076 (4)	−0.0223 (5)	0.0138 (5)
N3	0.0351 (5)	0.0412 (5)	0.0558 (7)	−0.0003 (4)	−0.0046 (5)	0.0017 (5)
N2	0.0354 (5)	0.0418 (6)	0.0534 (7)	−0.0037 (4)	−0.0030 (5)	0.0028 (5)
O4	0.0687 (7)	0.0791 (8)	0.0882 (9)	0.0000 (6)	−0.0168 (7)	−0.0363 (7)
O1	0.0631 (7)	0.0515 (6)	0.1188 (11)	−0.0111 (5)	−0.0220 (7)	−0.0053 (6)
N1	0.0451 (7)	0.0562 (7)	0.0783 (10)	−0.0071 (6)	−0.0122 (6)	−0.0018 (7)
C14	0.0327 (6)	0.0428 (7)	0.0492 (8)	0.0027 (5)	−0.0041 (6)	0.0002 (6)
C12	0.0360 (6)	0.0430 (7)	0.0451 (8)	−0.0008 (5)	−0.0008 (6)	0.0031 (6)
C13	0.0362 (6)	0.0389 (6)	0.0461 (8)	−0.0031 (5)	0.0003 (6)	0.0024 (6)
C7	0.0348 (6)	0.0467 (7)	0.0508 (8)	−0.0018 (5)	−0.0025 (6)	0.0013 (6)
C17	0.0450 (7)	0.0460 (7)	0.0481 (8)	−0.0002 (5)	−0.0044 (6)	0.0044 (6)
C8	0.0352 (6)	0.0478 (7)	0.0565 (9)	−0.0034 (5)	−0.0068 (6)	0.0020 (6)
C6	0.0382 (7)	0.0485 (7)	0.0511 (9)	0.0024 (5)	−0.0056 (6)	−0.0015 (6)
C11	0.0391 (7)	0.0487 (7)	0.0569 (9)	0.0006 (6)	−0.0046 (6)	0.0084 (6)
C16	0.0525 (8)	0.0448 (7)	0.0511 (9)	−0.0020 (6)	−0.0115 (7)	0.0009 (6)
C15	0.0424 (7)	0.0488 (7)	0.0579 (9)	−0.0040 (6)	−0.0137 (6)	0.0008 (7)
C18	0.0424 (7)	0.0526 (8)	0.0621 (10)	−0.0019 (6)	−0.0039 (7)	−0.0011 (7)
C9	0.0479 (8)	0.0384 (7)	0.0659 (10)	−0.0038 (6)	−0.0058 (7)	0.0021 (6)
C10	0.0482 (8)	0.0385 (7)	0.0719 (11)	0.0012 (6)	−0.0054 (7)	0.0077 (7)
C5	0.0432 (7)	0.0497 (8)	0.0680 (11)	0.0025 (6)	−0.0064 (7)	0.0001 (7)
C4	0.0622 (10)	0.0507 (8)	0.0727 (12)	0.0089 (7)	−0.0027 (8)	0.0057 (8)
C19	0.0520 (9)	0.0631 (9)	0.0780 (12)	−0.0102 (7)	−0.0117 (8)	−0.0004 (9)
C3	0.0594 (10)	0.0706 (10)	0.0704 (12)	0.0231 (8)	−0.0138 (8)	0.0037 (9)
C1	0.0421 (8)	0.0605 (9)	0.0871 (13)	−0.0009 (7)	−0.0118 (8)	0.0028 (8)
C22	0.0569 (9)	0.0647 (10)	0.0702 (12)	0.0109 (7)	−0.0026 (8)	−0.0061 (8)
C20	0.0408 (8)	0.0788 (11)	0.0959 (15)	−0.0031 (8)	−0.0076 (8)	0.0195 (11)
C2	0.0422 (8)	0.0765 (11)	0.0990 (15)	0.0076 (8)	−0.0172 (8)	−0.0003 (10)
C21	0.0534 (9)	0.0793 (12)	0.0880 (15)	0.0150 (9)	0.0052 (9)	0.0043 (10)

### Geometric parameters (Å, º)

**Table:**

O3—C14	1.3431 (16)	C15—H15A	0.9700
O3—C15	1.4365 (16)	C15—H15B	0.9700
O2—C14	1.2018 (16)	C18—C19	1.381 (2)
N3—C7	1.3491 (16)	C18—H18	0.9300
N3—C13	1.3526 (16)	C9—C10	1.3581 (19)
N2—C9	1.3729 (17)	C9—H9	0.9300
N2—C13	1.3918 (16)	C10—H10	0.9300
N2—C8	1.4092 (16)	C5—C4	1.386 (2)
O4—C16	1.2157 (17)	C5—H5	0.9300
O1—N1	1.2446 (16)	C4—C3	1.380 (2)
N1—C8	1.3732 (17)	C4—H4	0.9300
C14—C12	1.4951 (18)	C19—C20	1.385 (3)
C12—C11	1.3783 (18)	C19—H19	0.9300
C12—C13	1.4166 (18)	C3—C2	1.375 (3)
C7—C8	1.4115 (19)	C3—H3	0.9300
C7—C6	1.4803 (18)	C1—C2	1.381 (2)
C17—C18	1.390 (2)	C1—H1	0.9300
C17—C22	1.391 (2)	C22—C21	1.376 (2)
C17—C16	1.4907 (19)	C22—H22	0.9300
C6—C5	1.387 (2)	C20—C21	1.377 (3)
C6—C1	1.396 (2)	C20—H20	0.9300
C11—C10	1.4002 (19)	C2—H2	0.9300
C11—H11	0.9300	C21—H21	0.9300
C16—C15	1.511 (2)		
			
C14—O3—C15	117.98 (11)	H15A—C15—H15B	108.4
C7—N3—C13	105.94 (10)	C19—C18—C17	119.98 (15)
C9—N2—C13	123.04 (11)	C19—C18—H18	120.0
C9—N2—C8	131.29 (11)	C17—C18—H18	120.0
C13—N2—C8	105.67 (10)	C10—C9—N2	118.84 (12)
O1—N1—C8	117.36 (12)	C10—C9—H9	120.6
O2—C14—O3	124.53 (12)	N2—C9—H9	120.6
O2—C14—C12	125.28 (12)	C9—C10—C11	120.11 (13)
O3—C14—C12	110.15 (11)	C9—C10—H10	119.9
C11—C12—C13	118.33 (11)	C11—C10—H10	119.9
C11—C12—C14	120.41 (11)	C4—C5—C6	120.80 (14)
C13—C12—C14	120.94 (11)	C4—C5—H5	119.6
N3—C13—N2	111.88 (10)	C6—C5—H5	119.6
N3—C13—C12	130.17 (11)	C3—C4—C5	119.83 (15)
N2—C13—C12	117.93 (11)	C3—C4—H4	120.1
N3—C7—C8	111.24 (11)	C5—C4—H4	120.1
N3—C7—C6	121.04 (11)	C18—C19—C20	120.37 (16)
C8—C7—C6	127.70 (11)	C18—C19—H19	119.8
C18—C17—C22	119.06 (13)	C20—C19—H19	119.8
C18—C17—C16	122.36 (13)	C2—C3—C4	119.82 (14)
C22—C17—C16	118.53 (13)	C2—C3—H3	120.1
N1—C8—N2	127.33 (12)	C4—C3—H3	120.1
N1—C8—C7	127.34 (12)	C2—C1—C6	120.06 (16)
N2—C8—C7	105.26 (10)	C2—C1—H1	120.0
C5—C6—C1	118.72 (13)	C6—C1—H1	120.0
C5—C6—C7	119.55 (12)	C21—C22—C17	120.64 (16)
C1—C6—C7	121.73 (13)	C21—C22—H22	119.7
C12—C11—C10	121.69 (12)	C17—C22—H22	119.7
C12—C11—H11	119.2	C21—C20—C19	119.80 (15)
C10—C11—H11	119.2	C21—C20—H20	120.1
O4—C16—C17	121.74 (14)	C19—C20—H20	120.1
O4—C16—C15	118.84 (13)	C3—C2—C1	120.74 (15)
C17—C16—C15	119.41 (12)	C3—C2—H2	119.6
O3—C15—C16	108.54 (11)	C1—C2—H2	119.6
O3—C15—H15A	110.0	C22—C21—C20	120.14 (17)
C16—C15—H15A	110.0	C22—C21—H21	119.9
O3—C15—H15B	110.0	C20—C21—H21	119.9
C16—C15—H15B	110.0		
			
C15—O3—C14—O2	−14.9 (2)	C8—C7—C6—C1	23.0 (3)
C15—O3—C14—C12	163.13 (11)	C13—C12—C11—C10	2.5 (2)
O2—C14—C12—C11	141.14 (16)	C14—C12—C11—C10	−171.16 (15)
O3—C14—C12—C11	−36.82 (19)	C18—C17—C16—O4	177.35 (15)
O2—C14—C12—C13	−32.3 (2)	C22—C17—C16—O4	−5.1 (2)
O3—C14—C12—C13	149.69 (13)	C18—C17—C16—C15	−3.6 (2)
C7—N3—C13—N2	0.14 (16)	C22—C17—C16—C15	173.89 (14)
C7—N3—C13—C12	−178.13 (15)	C14—O3—C15—C16	−89.28 (14)
C9—N2—C13—N3	−179.58 (13)	O4—C16—C15—O3	19.49 (19)
C8—N2—C13—N3	0.31 (16)	C17—C16—C15—O3	−159.56 (12)
C9—N2—C13—C12	−1.1 (2)	C22—C17—C18—C19	−1.0 (2)
C8—N2—C13—C12	178.82 (12)	C16—C17—C18—C19	176.52 (14)
C11—C12—C13—N3	176.84 (15)	C13—N2—C9—C10	2.4 (2)
C14—C12—C13—N3	−9.5 (2)	C8—N2—C9—C10	−177.50 (15)
C11—C12—C13—N2	−1.3 (2)	N2—C9—C10—C11	−1.2 (2)
C14—C12—C13—N2	172.27 (13)	C12—C11—C10—C9	−1.2 (3)
C13—N3—C7—C8	−0.56 (17)	C1—C6—C5—C4	−1.3 (2)
C13—N3—C7—C6	−179.33 (13)	C7—C6—C5—C4	178.39 (15)
O1—N1—C8—N2	2.6 (2)	C6—C5—C4—C3	0.2 (3)
O1—N1—C8—C7	179.29 (16)	C17—C18—C19—C20	0.4 (2)
C9—N2—C8—N1	−3.5 (3)	C5—C4—C3—C2	1.0 (3)
C13—N2—C8—N1	176.62 (15)	C5—C6—C1—C2	1.2 (3)
C9—N2—C8—C7	179.26 (15)	C7—C6—C1—C2	−178.44 (16)
C13—N2—C8—C7	−0.61 (15)	C18—C17—C22—C21	0.7 (2)
N3—C7—C8—N1	−176.49 (15)	C16—C17—C22—C21	−176.95 (16)
C6—C7—C8—N1	2.2 (3)	C18—C19—C20—C21	0.4 (3)
N3—C7—C8—N2	0.74 (17)	C4—C3—C2—C1	−1.0 (3)
C6—C7—C8—N2	179.42 (14)	C6—C1—C2—C3	−0.1 (3)
N3—C7—C6—C5	21.9 (2)	C17—C22—C21—C20	0.2 (3)
C8—C7—C6—C5	−156.69 (16)	C19—C20—C21—C22	−0.8 (3)
N3—C7—C6—C1	−158.46 (15)		

### Hydrogen-bond geometry (Å, º)

*Cg*4 is the centroid of the C17–C22 phenyl ring.

**Table:**

*D*—H···*A*	*D*—H	H···*A*	*D*···*A*	*D*—H···*A*
C15—H15*A*···O4^i^	0.97	2.54	3.1257 (19)	119
C15—H15*B*···O1^ii^	0.97	2.61	3.4841 (18)	150
C9—H9···O2^iii^	0.93	2.46	3.1176 (16)	128
C10—H10···O2^iii^	0.93	2.67	3.2243 (17)	119
C9—H9···O1	0.93	2.35	2.8736 (18)	116
C1—H1···N1	0.93	2.51	3.081 (2)	120
C22—H22···*Cg*4^iv^	0.93	2.80	3.657 (2)	153

Symmetry codes: (i) *x*, −*y*+1/2, *z*+1/2; (ii) −*x*+1, *y*+1/2, −*z*+1/2; (iii) −*x*+1, *y*−1/2, −*z*+1/2; (iv) *x*, −*y*+1/2, *z*−1/2.

## References

[bb1] Anaflous, A., Albay, H., Benchat, N., El Bali, B., Dušek, M. & Fejfarová, K. (2008). *Acta Cryst.* E**64**, o926. 10.1107/S1600536808011501 PMC2961311 21202407

[bb2] Bode, M. L., Gravestock, D., Moleele, S. S., van der Westhuyzen, C. W., Pelly, S. C., Steenkamp, P. A., Hoppe, H. C., Khan, T. & Nkabinde, L. A. (2011). *Bioorg. Med. Chem.* **19**, 4227–4237. 10.1016/j.bmc.2011.05.062 21700466

[bb3] Damghani, T., Hadaegh, S., Khoshneviszadeh, M., Pirhadi, S., Sabet, R., Khoshneviszadeh, M. & Edraki, N. (2020). *J. Mol. Struct.* **1222**, 128876.

[bb4] Daoui, S., Cinar, E. B., Dege, N., Chelfi, T., El Kalai, F., Abudunia, A., Karrouchi, K. & Benchat, N. (2021). *Acta Cryst.* E**77**, 23–27. 10.1107/S205698902001573X PMC7784059 33520277

[bb5] Daoui, S., Muwafaq, I., Çınar, E. B., Abudunia, A., Dege, N., Benchat, N. & Karrouchi, K. (2022). *Acta Cryst.* E**78**, 8–11. 10.1107/S205698902101238X PMC8739192 35079414

[bb6] Deep, A., Bhatia, R. K., Kaur, R., Kumar, S., Jain, U. K., Singh, H., Batra, S., Kaushik, D. & Deb, P. K. (2017). *Curr. Top. Med. Chem.* **17**, 238–250. 10.2174/1568026616666160530153233 27237332

[bb7] Elaatiaoui, A., Saddik, R., Benchat, N., Saadi, M. & El Ammari, L. (2015). *Acta Cryst.* E**71**, o803–o804. 10.1107/S2056989015017843 PMC4647394 26594488

[bb8] Farrugia, L. J. (2012). *J. Appl. Cryst.* **45**, 849–854.

[bb9] Groom, C. R., Bruno, I. J., Lightfoot, M. P. & Ward, S. C. (2016). *Acta Cryst.* B**72**, 171–179. 10.1107/S2052520616003954 PMC4822653 27048719

[bb10] Gundlewad, G. B., Wagh, S. S. & Patil, B. R. (2020). *Asia. J. Org. Med. Chem.* **5**, 221–226.

[bb11] El Kalai, F., Çınar, E. B., Lai, C. H., Daoui, S., Chelfi, T., Allali, M., Dege, N., Karrouchi, K. & Benchat, N. (2021*a*). *J. Mol. Struct.* **1228**, 129435. 10.1016/j.molstruc.2020.129435 PMC7546970 33071353

[bb12] El Kalai, F., Karrouchi, K., Baydere, C., Daoui, S., Allali, M., Dege, N., Benchat, N. & Brandán, S. A. (2021*b*). *J. Mol. Struct.* **1223**, 129213.

[bb13] Macrae, C. F., Sovago, I., Cottrell, S. J., Galek, P. T. A., McCabe, P., Pidcock, E., Platings, M., Shields, G. P., Stevens, J. S., Towler, M. & Wood, P. A. (2020). *J. Appl. Cryst.* **53**, 226–235. 10.1107/S1600576719014092 PMC6998782 32047413

[bb14] Mishra, N. P., Mohapatra, S., Sahoo, C. R., Raiguru, B. P., Nayak, S., Jena, S. & Padhy, R. N. (2021). *J. Mol. Struct.* **1246**, 131183.

[bb15] Saeedi, M., Raeisi-Nafchi, M., Sobhani, S., Mirfazli, S. S., Zardkanlou, M., Mojtabavi, S., Faramarzi, M. A. & Akbarzadeh, T. (2021). *Mol. Divers.* **25**, 2399–2409. 10.1007/s11030-020-10137-8 33047276

[bb16] Sheldrick, G. M. (2015*a*). *Acta Cryst.* A**71**, 3–8.

[bb17] Sheldrick, G. M. (2015*b*). *Acta Cryst.* C**71**, 3–8.

[bb18] Sigalapalli, D. K., Kiranmai, G., Parimala Devi, G., Tokala, R., Sana, S., Tripura, C., Jadhav, G. S., Kadagathur, M., Shankaraiah, N., Nagesh, N., Babu, B. N. & Tangellamudi, N. D. (2021). *Bioorg. Med. Chem.* **43**, 116277. 10.1016/j.bmc.2021.116277 34175586

[bb19] Spek, A. L. (2020). *Acta Cryst.* E**76**, 1–11. 10.1107/S2056989019016244 PMC6944088 31921444

[bb20] Stoe & Cie (2012). *X-AREA* and *X-RED32*. Stoe & Cie GmbH, Darmstadt, Germany.

[bb21] Swainston Harrison, T. & Keating, G. M. (2005). *CNS Drugs*, **19**, 65–89. 10.2165/00023210-200519010-00008 15651908

[bb22] Turner, M. J., McKinnon, J. J., Wolff, S. K., Grimwood, D. J., Spackman, P. R., Jayatilaka, D. & Spackman, M. A. (2017). *CrystalExplorer17. University of Western Australia.* http://hirshfeldsurface.net.

[bb23] Wang, A., Lv, K., Li, L., Liu, H., Tao, Z., Wang, B., Liu, M., Ma, C., Ma, X., Han, B., Wang, A. & Lu, Y. (2019). *Eur. J. Med. Chem.* **178**, 715–725. 10.1016/j.ejmech.2019.06.038 31229874

[bb24] Westrip, S. P. (2010). *J. Appl. Cryst.* **43**, 920–925.

[bb25] Yu, Y. N., Han, Y., Zhang, F., Gao, Z., Zhu, T., Dong, S. & Ma, M. (2020). *J. Med. Chem.* **63**, 3028–3046. 10.1021/acs.jmedchem.9b01736 32069401

